# Expiration of the Pylorus

**Published:** 1881-06

**Authors:** 


EXTIRPATION OF THE PYLORUS.

"We to-day register a triumph which surgical art has recently been enabled to celebrate. The important question which has so long engaged the attention of surgeons—whether the carcinoma which so frequently attacks the stomach, and for which all internal remedies are useless, may not be made amenable to operative surgery—would seem to be at once decided affirmatively. On January 29, Prof. Billroth performed a partial excision of the stomach on account of advanced carcinoma of the pylorus, which up to the present time has been attended with success. Already, seventy years ago, Karl Merrem had shown in a dissertation, by experiments upon dogs, that the pylorus might be excised, and recommended the procedure in incurable cancer. The conviction, however, that the vital processes in man and animals were the same, was as yet too little assured, and operative procedures too little advanced, for the operation to be ventured on. It is only during the last ten years that essential progress has been made in this province of surgery; and Billroth and his pupils have done much in that time to prepare the way for the operation. After Billroth had shown, in 1871, that portions of the esophagus may be cut out in dogs, Czerny first performed this operation on man. Then followed Gussenbauer's and Winniwarter's excision of portions of the intestinal canal and stomach in dogs, and Martini and Gussenbauer's successful excision of a sigmoid flexure that had undergone cancerous degeneration. Gastrorraphy, successfully performed by Billroth in 1877, was another step in advance, and led him to make the remark that from this operation to the excision of a cancerous portion of the stomach required but one bold step. The first, however, who performed excision (in 1829) for cancerous pylorus in man was the Parisian surgeon Pean, so well known for his numerous leparotomies, but the patient died on the fourth day; and no suitable case came under Billroth's notice until the present one.

"The woman upon whom the operation was performed on January 29, was forty-three years of age, and previously in good health, having borne eight children. In October, 1880, she suffered from vomiting, and soon presented all the symptoms of carcinoma of the stomach and stenosis of the pylorus. During the six weeks prior to the operation the constant vomiting and small amount of nourishment taken led to excessive pallor, emaciation, small and frequent pulse, and exhaustion; so that the patient, feeling her end approaching, consented to the operation proposed by Billroth. The preparation for the operation, which was performed in a temperature of 24° R. (86° Fahr.) under chloroform, consisted only in washing out the stomach with the ordinary tube. An incision about eight centimetres in length was performed over the tumor, which was readily movable under the thinned integuments. The tumor proved to be a nodulated carcinoma of the pylorus, which was in part infiltrated, and occupied more than the lower third of the stomach. The parts were carefully separated from the omentum and transverse colon, and, the vessels being tied before their division, very little blood was lost. The tumor having been completely brought on to the integuments of the abdomen, an incision was made through the stomach one centimetre beyond the infiltrated part—first only backwards, and then through the duodenum. An oblique incision through the stomach was then directed from above and inwards to below and outwards, always at a distance of one centimetre from the infiltrated parts. After unitting the oblique incision only sufficiently to allow of its being adjusted to the duodenum, the tumor was completely separated from the duodenum, one centimetre from the infiltration, by means of an incision parallel to that made in the stomach. The duodenum was adapted to the aperture left in the stomach, about fifty sutures of carbolized silk in all having been employed during the operation. After cleansing with a two per cent. carbolic acid solution, and the application of a guard-ligature, the parts were replaced in the cavity of the abdomen. The operation, including a tedious chloroformization, occupied one hour and a half. The excised portion consisted of fourteen centimetres of the greater curvature of the stomach, and a quill could only be passed with difficulty through the pylorus. The form of the stomach was not essentially changed by the operation, the organ only being rendered smaller. After the operation there was neither vomiting nor pain. * * * From a communication of Prof. Billroth on February 13, it appears that the patient had continued to improve, so that the recovery then seemed assured. The result is indeed favorable beyond all expectation, and already suffices to show that such an operation is practicable, so that persons may now be successfully treated for a disease hitherto reputed incurable; and even when a relapse takes place, they will at least have received temporary alleviation."—Prof. Billroth in *Wiener Med. Woch.*

